# The association between dietary intake of flavonoids and its subclasses and the risk of metabolic syndrome

**DOI:** 10.3389/fnut.2023.1195107

**Published:** 2023-07-05

**Authors:** Zhenlei Zhao, Wenyan Gao, Xiaoli Ding, Xiaogang Xu, Changqian Xiao, Genxiang Mao, Wenmin Xing

**Affiliations:** ^1^Zhejiang Provincial Key Lab of Geriatrics, Zhejiang Hospital, Hangzhou, China; ^2^School of Pharmacy, Hangzhou Medical College, Hangzhou, China

**Keywords:** metabolic syndrome, flavonoids intake, flavanones, anthocyanidins, NHANES

## Abstract

**Background:**

The healthiest way to prevent metabolic syndrome (MetS) is through behavioral and nutritional adjustments. We examined the relationship between total flavonoids intake, flavonoid subclasses, and clinically manifest MetS.

**Methods:**

A cross-sectional analysis was conducted among 28,719 individuals from the National Health and Nutrition Examination Survey (NHANES) and Food and Nutrient Database for Dietary Studies (FNDDS) 2007–2011 and 2017–2018. Two 24-h reviews were conducted to determine flavonoids intake and subclasses. The link between flavonoids intake and MetS was investigated using a multivariate logistic regression model.

**Results:**

Q2 and Q3 of total flavonoids intake were associated with 20 and 19% lower risk of incident MetS after adjusting age and sex. Anthocyanidins and flavanones intake in Q2 and Q3 substantially reduced the MetS risk compared to Q1. MetS risk decreased steadily as the total intake of flavonoids increased to 237.67 mg/d. Flavanones and anthocyanidins also displayed V-shaped relationship curves (34.37 and 23.13 mg/d).

**Conclusion:**

MetS was adversely linked with total flavonoids intake, flavanones, and anthocyanidins. Moreover, the most effective doses of total flavonoids, flavanones, and anthocyanidins were 237.67, 34.37, and 23.13 mg/d, respectively, potentially preventing MetS.

## Introduction

Metabolic syndrome (MetS) is characterized by a cluster of metabolic abnormalities, such as impaired glucose metabolism, high blood pressure, low high-density lipoprotein cholesterol (HDL-c) levels, and dyslipidemia (1). Subjects with MetS mainly present with abdominal obesity, hyperglycemia, hypertension, and dyslipidemia (2). It was also closely associated with a higher cardiovascular disease (CVD) risk, type 2 diabetes (T2D) risk, and overall mortality (3, 4). MetS results from complicated risk factors interaction among genetic, metabolic, diet, lifestyle, and environmental factors (5). For instance, smoking, alcohol drinking, and an unbalanced diet contributed significantly to the development of MetS (5). Studies have demonstrated that some dietary life and nutrients played a protective role against MetS development (6, 7). For instance, Mediterranean diet (MedDiet) interventions could improve MetS (8, 9). MedDiet is characterized by a high concentration of polyphenols, which are prospective candidates for ameliorating the chronic low-grade inflammation and oxidative stress of MetS patients (10).

Previous research examined the relationship between dietary polyphenols and MetS, CVD, T2D, cancer, and all-cause mortality (11–17). For instance, high total polyphenols, flavonoids, and phenolic acid intake had 50, 51, and 45% lower odds of MetS, respectively, when compared to low total polyphenols, flavonoids, and phenolic acid intake. In contrast, larger intakes of total polyphenols, flavonoids, and phenolic acids were related to a reduced risk for elevated systolic blood pressure (SBP) and HDL-c, which were independent cardiovascular risk factors (16). However, no relationship was found between total polyphenols intake and other MetS components (18). A French prospective cohort study reported that individuals with polyphenols intake, including anthocyanins, dihydro flavonols, catechins, flavonols, hydroxybenzoic acids, lignans, and stilbenes, had a low risk of T2D, which is independent of major potential confounders (11). Furthermore, a decreased risk of gestational diabetes mellitus (GDM) is related to a large intake of fruit polyphenols. However, no clear association exists between total vegetable polyphenol intake and GDM risk (15). Moreover, a high intake of stilbenes, lignans, hydroxy benzaldehydes, hydroxy coumarins, and tyrosols was associated with a lower gastric cancer risk (12). Additionally, peonidin, naringenin, and catechin intakes were negatively correlated with cancer mortality (17). However, total polyphenols intake was not significantly associated with cancer mortality risk reported in Japanese adults (13). Importantly, individuals' biological aging or the discrepancy between the biological and chronological age of a subject (Δage) was inversely associated with the polyphenol antioxidant content (PAC) score (14). Therefore, a polyphenol-rich diet helps decelerate biological aging, which benefits the long-term risk of MetS, cardiovascular disease, and cancer.

Previous studies have highlighted dietary flavonoids intake as a potential protective factor against developing extra body fat (19). Studies have evidenced that flavonoids are strong antioxidants and metal chelators to decrease energy intake, increase energy expenditure and fat oxidation, influence macronutrient absorption and uptake, and inhibit adipogenesis (20–25). According to a previous study, long-term use of flavonoids may improve cardiovascular disease by restoring the body's natural antioxidant defenses and lowering the risk of low-density lipoprotein cholesterol (LDL) oxidation (26). In addition, flavonoid compounds extracted from apple peel can reduce blood pressure and control body fat (27, 28). Although a previous cross-sectional study including 9,108 Chinese individuals investigated the relationship between flavonoid and copper intake and MetS, there is no proof that any flavonoid compound can ameliorate MetS symptoms (5). A Tehran Lipid and Glucose Study based on Iran's population reported that only flavonols and flavones showed protective effects against MetS. Simultaneously, no significant association was found between the intake of other flavonoid subclasses and MetS risk (29). Due to the intricacy of MetS and the variety of flavonoids, additional research is required to assess the relationship between flavonoid consumption and MetS risk. Furthermore, because each flavonoid component has unique active activity, it was necessary to confirm whether consuming the various flavonoid subclasses was linked with lowering MetS (18).

Consequently, this study aimed to demonstrate the possible association of total flavonoids and subclasses of flavonoid intake with MetS in the USA using the NHANES data system from 2007 to 2010 and 2017 to 2018. We hypothesized that flavonoids intake would be positively associated with a reduced risk of MetS. Additionally, the impact of an individual's clinical characteristics and lifestyle on the MetS reduction brought on by using flavonoids was discussed. This study also explored the most effective dose of each flavonoid subtype for reducing the MetS rate.

## Methods

### Study population

The NHANES was a cross-sectional design that employed stratified, multistage probability sampling of the U.S. population to examine health and nutritional status through interviews and laboratory examinations (30). This survey was approved by the Ethics Review Board of the NCHS (available on the web at: https://www.cdc.gov/nchs/nhanes/). This study employed three cycles of NHANES 2007–2010 and 2017–2018 data to extract flavonoids intake information, and 28,719 participants were included (Table 1). Participants were disqualified if they could not provide dietary information within 24 h. Moreover, pregnant women and cancer patients receiving medical therapy or radiotherapy were excluded. Demographic, health-related lifestyle, and chronic disease information were also gathered from the participants.

**Table 1:** Characteristics of NHANES participants by tertiles of total flavonoid intake.

	**Total population (*n =* 28,719)**	**Total flavonoid intake quintiles**			
		**Q1 (*n =* 8,937)**	**Q2 (*n =* 9,265)**	**Q3 (*n =* 10,517)**	***P*-value**
Total flavonoid intake, mean (SE) (mg/d)	220.03 (7.37)	18.57 (0.34)	80.00 (0.93)	579.24 (13.69)	< 0.0001
**Socio-economic characteristics**					
Female, *n* (%)	14,690 (51.15)	4,553 (50.95)	4,656 (50.25)	3,851 (52.10)	0.21
Male, *n* (%)	14,029 (48.85)	4,384 (49.05)	4,609 (49.75)	5,409 (47.90)	
Age, years, mean (SE)	37.26 (0.29)	33.62 (0.39)	37.17 (0.39)^a*^	40.44 (0.41)^b*^	< 0.0001
**Ethnicity**					
White, *n* (%)	1,8191 (63.34)	5,656 (63.29)	5,716 (61.69)^a*^	6,820 (64.85)^b*^	< 0.0001
Black, *n* (%)	3,464 (12.06)	1,219 (13.64)	1,106 (11.94)^a*^	1,140 (10.84)^b*^	
Mexican, *n* (%)	2,970 (10.34)	990 (11.08)	1,061 (11.45)^a*^	919 (8.74)^b*^	
Others, *n* (%)	4,092 (14.25)	1,072 (12.00)	1,383 (14.93)^a*^	1,639 (15.58)^b*^	
**Healthy behavior factors**					
Smoke status					
Current smokers, *n* (%)	4,431 (15.43)	1,635 (18.30)	1,141 (12.32)^a*^	1,54 (15.73)^b*^	< 0.0001
No current smokers, *n* (%)	24,288 (84.57)	7,302 (81.70)	8,124 (87.68)^a*^	8,863 (84.27)^b*^	
**Drinking**					
No drink user, *n* (%)	6,979 (24.30)	2,308 (25.83)	2,339 (25.25)^a*^	2,330 (22.15)^b*^	< 0.0001
Former drink user, *n* (%)	2,257 (7.86)	798 (8.93)	671 (7.24)^a*^	788 (7.49)^b*^	
Mild drink user, *n* (%)	8,587 (29.90)	2,166 (24.24)	2,867 (30.94)^a*^	3,555 (33.80)^b*^	
Moderate drink user, *n* (%)	4,474 (15.58)	1,423 (15.92)	1,390 (15.00)^a*^	1663 (15.81)^b*^	
Heavy drink user, *n* (%)	6,422 (22.36)	2,241 (25.07)	1,998 (21.57)^a*^	2,181 (20.74)^b*^	
**Physical activity level**					
Never, *n* (%)	10,399 (36.21)	3,695 (41.34)	3,327 (35.91)^a*^	3,377 (32.11)^b*^	< 0.0001
Low, *n* (%)	5,775 (20.11)	1,589 (17.78)	1,885 (20.35)^a*^	2,301 (21.88)^b*^	
Intermediate, *n* (%)	6,304 (21.95)	1,723 (19.28)	2,129 (22.98)^a*^	2,453 (23.32)^b*^	
High, *n* (%)	6,241 (21.73)	1,930 (21.60)	1,924 (20.77)^a*^	2,386 (22.69)^b*^	
**Dietary intake**					
kCal/day, kCal, mean (SE)	2,032.07 (8.20)	1,846.08 (11.69)	2,091.19 (12.00)^a*^	2,138.07 (12.75)^b*^	< 0.0001
Carbohydrates/day, g/100 kCal, mean (SE)	249.00 (0.95)	222.01 (1.69)	257.74 (1.44)^a*^	264.23 (1.54)^b*^	< 0.0001
Protein/day, g/100 kCal, mean (SE)	78.01 (0.40)	71.10 (0.57)	80.17 (0.54)^a*^	81.99 (0.51)^b*^	< 0.0001
Fiber/day, g/100 kCal, mean (SE)	15.85 (0.15)	12.32 (0.13)	17.25 (0.14)^a*^	17.61 (0.19)^b*^	< 0.0001
Total fat, mean (SE)	78.49 (0.39)	73.27 (0.54)	79.01 (0.60)^a*^	82.47 (0.60)^b*^	< 0.0001
**Medications**, ***n*** **(%)**					
Anti.Diabetic					
No	26,829 (93.42)	8,344 (93.37)	8,687 (93.76)	9,798 (93.16)^b*^	0.6
Yes	1,890 (6.58)	593 (6.63)	578 (6.24)	719 (6.84)^b*^	
Anti.Hypertensive					
No	27,533 (95.87)	8,628 (96.54)	8,866 (95.69)	10,041 (95.47)^b*^	0.08
Yes	1,186 (4.13)	309 (3.46)	399 (4.31)	476 (4.53)^b*^	
BMI, kg/m^2^, *n* (%)					
BMI < 25	12,677 (44.14)	4,027 (45.06)	4,260 (45.98)	4,390 (41.74)	0.001
BMI ≥ 25	16,042 (55.86)	4,910 (54.94)	5,005 (54.02)	6,127 (58.26)	
**Hypertension**, ***n*** **(%)**					
No Hypertension	20,816 (72.48)	6,638 (74.28)	6,755 (72.91)	7,421 (70.56)^b*^	< 0.0001
Hypertension	7,903 (27.52)	2,299 (25.72)	2,510 (27.09)	3,096 (29.44)^b*^	
**Diabetes**, ***n*** **(%)**					
No diabetes	23,972 (83.47)	7,529 (84.25)	7,759 (83.75)	8,683 (82.56)	0.11
Diabetes	4,747 (16.53)	1,408 (15.75)	1,506 (16.25)	1,834 (17.44)	
**Chronic kidney disease**					
No CKD	20,867 (72.66)	6,212 (69.51)	6,523 (70.40)	8,132 (77.32)^b*^	< 0.0001
CKD	7,852 (27.34)	2,725 (30.49)	2,742 (29.60)	2,385 (22.68)^b*^	
**COPD**					
No COPD	27,645 (96.26)	8,593 (96.15)	8,931 (96.39)	10,122 (96.24)	0.82
COPD	1,074 (3.74)	344 (3.85)	334 (3.61)	395 (3.76)	
**CVD**					
No CVD	26,855 (93.51)	8,375 (93.71)	8,707 (93.98)	9,773 (92.93)	0.03
CVD	1,864 (6.49)	562 (6.29)	558 (6.02)	744 (7.07)	
**MetS**					
No MetS	22,694 (79.02)	6,995 (78.27)	7,417 (80.05)^a*^	8,280 (78.73)	0.15
MetS	6,025 (20.98)	1,942 (21.73)	1,848 (19.95)^a*^	2,237 (21.27)	

Values are means (SE) for continuous variables and *n* (%) for categorical variables; SE, standard error; NHANES, National Health and Nutrition Examination Survey; BMI, body mass index; CKD, chronic kidney disease; MET, metabolic equivalent; COPD, chronic obstructive pulmonary disease; CVD, cardiovascular disease; MetS, metabolic syndrome. The total flavonoid value was the sum of 29 kinds of flavonoids in six subclasses of classes, which include anthocyanidins, flavan-3-ols, flavanones, flavones, flavonols, and isoflavones. Data were generated using χ^2^ tests for the categorical variables, and *T*-tests were used for continuous variables. ^a*^indicated that the *p*-value for the Q2 vs. Q1 comparison was < 0.05, while ^b*^indicated that the *p*-value for the Q3 vs. Q1 comparison was < 0.05.

### Assessment of flavonoid intakes

The information on total flavonoids and their subclasses intake was extracted from the United States Department of Food and Nutrient Database for Dietary Studies (FNDDS) linked to the NHANES database. These flavonoid compounds' levels were determined by averaging the results of two 24-h interviews (31). The USDA food code for each survey cycle was used to assign the flavonoid compounds (version 4.1 for 2007–2008 and version 5.0 for 2009–2010) (32). USDA Database provided values for 29 kinds of flavonoids, six flavonoid subclasses (anthocyanidins, flavan-3-ols, flavanones, flavones, flavonols, and isoflavones), and total flavonoids for all food codes linked to NHANES 2007–2010 and 2017–2018 (32). These values can be used to estimate flavonoids consumption in the U.S. population.

### Definition of metabolic syndrome

Metabolic syndrome (MetS) is defined according to the criteria and definition published in the Lancet statement on metabolic syndrome in 2005 guidelines (33, 34). The following requirements were described in detail: (1) waist circumference was ≥88 cm for women and ≥102 cm for men, (2) hypertriglyceridemia (triglycerides ≥ 1.7 mmol/L), (3) low HDL cholesterol (HDL < 1.03 mmol/L in men or HDL < 1.29 mmol/L in women), (4) elevated blood pressure (SBP ≥ 130 mm Hg, DBP ≥ 85 mm Hg, or both) or antihypertensive drug treatment for hypertension, and (5) elevated fasting plasma glucose (FPG) (FPG ≥ 5.6 mmol/L, or diagnosed as type 2 diabetes).

### Covariates

Using standard questionnaires, NHANES supplied demographic and lifestyle information about people in this study. In this study, gender, age, ethnicity, and body mass index (BMI) were demographic variables. Furthermore, smoking, drinking, physical activity, and fiber or protein consumption were lifestyle variables. Smoking was classified as no current (those who had never smoked more than 100 cigarettes in their lifetime or had smoked at least 100 cigarettes but did not currently smoke) or current (a minimum of 100 cigarettes had been smoked, or had smoked some days). Alcohol consumers were categorized as nondrinkers, previous drinkers, light, moderate, and heavy. Physical activity was evaluated by self-report and measured in weekly metabolic equivalent (MET) minutes. MET was calculated into trisection [Q1 (low), Q2 (intermediate), and Q3 (high)], and participants were categorized as never, low, intermediate, and high levels of physical activity. The ethnicity categories were White, Black, Mexican, and other ethnicity. Hypertension, diabetes, chronic kidney disease, and COPD were defined. Medication information, including anti-diabetes and anti-hypertension drugs, was defined as “No” and “Yes”.

### Statistical analyses

We used NHANES-recommended weights to balance for planned oversampling and ensure the analysis accuracy. The continuous variables were expressed as means ± standard errors (SE). Categorical variables were expressed as counts and percentages. Moreover, missing data were imputed using the forest R package. Individuals were separated into three groups based on total flavonoids and flavonoid subclasses' consumption values (tertiles: Q1, Q2, and Q3). Significant differences between MetS subjects and control subjects were identified using χ^2^ tests for categorical variables and ANOVA analysis for continuous variables. To evaluate the adjusted odds ratios (OR) and 95% confidence intervals (CIs) between flavonoids intake and MetS risk, a multivariate logistic regression analysis model was used. A model-adjusted risk ratio was calculated to compare the risk of MetS and flavonoids intake (Q2 and Q3) with the lowest (Q1) category. Then, we also constructed several adjusted models to modify various characteristics: model 1 (adjusted for age), model 2 (adjusted for age, sex, and ethnicity), model 3 (Model 2 plus smoke status, drinking status, and physical activity), and model 4 (Model 2 plus BMI, hypertension, diabetes, COPD, and medications). Then, a restricted cubic spline (RCS) was employed to explore the non-linear associations between the risk of MetS and the total flavonoids and its subclasses intake (35). A stratified analysis was performed to explore the heterogeneity of the effect of flavonoids intake on MetS risk. Moreover, an interaction model was used to evaluate the interaction between flavonoids intake and other variables. In this study, *p*-values of < 0.05 were considered statistically significant. R software (version 4.1.2), Rstudio software, and the nhanesR package were used for all analyses.

## Results

### Basic characteristics of this study population

In 2007–2010 and 2017–2018, for all included individuals, the mean (SE) total flavonoids intake in the first tertile was 18.57 (0.34) mg, in the second tertile was 80.00 (0.93) mg, and in the third tertile was 579.24 (13.69) mg. The demographic, lifestyle characteristics, dietary intake components, and diseases of the included individuals are illustrated in Table 1. This population of 28,719 individuals from the USA was followed for a mean age of 37.26 years, and 14,029 (48.85%) were men. According to the criteria, 6,025 people (20.98%) were identified as MetS participants. Compared to participants in Q1 of total flavonoids intake, those in Q3 were more likely to be older, have a lower BMI, and be more physically active. Participants who consumed more flavonoids also tended to consume more total fat, protein, carbs, and fiber. Furthermore, these individuals take more anti-hypertension drugs (Table 1). These participants were less likely to be smokers and heavy drink users with CKD and COPD. However, these participants had a higher risk of diabetes, hypertension, and CVD (Table 1).

### Overall associations between total flavonoids and six subclassess intake and MetS

We classified the total flavonoids intake by tertiles to investigate the association between flavonoids and MetS. The mean (SE) values of total flavonoids, six subclasses of flavonoids, and sole flavonoids are listed in Supplementary material 1. A multiple logistic regression model indicated that the Q2 and Q3 of total flavonoids intake were associated with a 20 and 19% lower risk of incident MetS after adjusting age and sex (model 1: second vs. first tertile, OR = 0.80 (95% CI: 0.71–0.90); Q3 vs. Q1, OR = 0.81 (95% CI: 0.72–0.91), P trend = 0.001) (Table 2). Total flavonoids intake is still strongly inversely associated with the MetS risk after adjusting for age, sex, lifestyles, and other nutrient intakes (Model 2: second vs. first tertile, OR = 0.82 (95% CI: 0.73–0.92); Q3 vs. Q1, OR = 0.83 (95% CI: 0.74–0.93), P trend = 0.003; model 3: Q2 vs. Q1, OR = 0.87 (95% CI: 0.76–0.98); Q3 vs. Q1, OR = 0.85 (95% CI: 0.75–0.97), P trend = 0.018). However, there were no statistically significant differences between the flavonols and flavones intake and the MetS risk. There were significant differences between the flavan_3_ols intake and the MetS risk only in model 1 (adjusting for sex and age) and model 2 (adjusting for sex, age, and lifestyles). The analysis showed that Q2 of flavanones intake reduced the risk of MetS compared to Q1 in model 1 (OR = 0.80, 95%CI: 0.71–0.89), model 2 (OR = 0.80, 95% CI: 0.71–0.89), and model 3 (OR = 0.84, 95% CI: 0.79–0.95). Furthermore, Q3 of flavanones intake had more strongly reduced the MetS risk than Q1 (model 1: OR = 0.73, 95% CI: 0.66–0.80), model 2 (OR = 0.73, 95% CI: 0.66–0.81), and model 3 (OR = 0.75, 95% CI: 0.67–0.84). Individuals who consumed anthocyanidins also experienced a considerable reduction in their MetS risk, as indicated in Table 2.

**Table 2:** Odd ratios (ORs) and 95% confidence intervals (CIs) of MetS risk by tertiles of flavonoid intake.

	**Total flavonoid intake quintiles**			***P* for trend**
**No. events**	**Q1 (*n =* 8,937)**	**Q2 (*n =* 9,265)**	**Q3 (*n =* 10,517)**	
**Total flavonoid**				
Mean (SE) (mg/d)	18.57 (0.34)	80.00 (0.93)	579.24 (13.69)	< 0.0001
**OR (95%CI)**				
Model 1	Ref	0.80 (0.71,0.90)	0.81 (0.72,0.91)	0.001
Model 2	Ref	0.82 (0.73,0.92)	0.83 (0.74,0.93)	0.003
Model 3	Ref	0.87 (0.76,0.98)	0.85 (0.75,0.97)	0.018
**Flavonols**				
No. events	8,136	9,026	11,557	
Mean (SE) (mg/d)	4.29 (0.03)	11.65 (0.04)	26.85 (0.33)	< 0.0001
**OR (95%CI)**				
Model 1	Ref	0.83 (0.74,0.93)	0.95 (0.85,1.07)	0.722
Model 2	Ref	0.82 (0.73,0.92)	0.95 (0.84,1.07)	0.696
Model 3	Ref	0.87 (0.77,0.98)	1.07 (0.97,1.21)	0.145
**Flavan_3_ols**				
No. events	9,121	9,268	10,327	
Mean (SE) (mg/d)	4.12 (0.05)	39.23 (0.88)	351.61 (9.93)	< 0.0001
**OR (95%CI)**				
Model 1	Ref	0.82 (0.74,0.92)	0.88 (0.798,0.97)	0.02
Model 2	Ref	0.84 (0.75,0.94)	0.89 (0.81,0.98)	0.041
Model 3	Ref	0.89 (0.80,1.00)	0.91 (0.81,1.01)	0.077
**Flavones**				
No. events	8,570	9,101	11,048	
Mean (SE) (mg/d)	0.10 (0.00)	0.51 (0.00)	1.54 (0.04)	< 0.0001
**OR (95%CI)**				
Model 1	Ref	0.91 (0.81,1.01)	0.96 (0.84,1.09)	0.064
Model 2	Ref	0.87 (0.78,0.97)	0.8793 (0.78,0.99)	0.059
Model 3	Ref	0.89 (0.79,0.98)	0.8855 (0.78,0.99)	0.582
**Flavanones**				
No. events	9,922	9,943	8,854	
Mean (SE) (mg/d)	0.06 (0.00)	7.34 (0.15)	36.55 (0.50)	< 0.0001
**OR (95%CI)**				
Model 1	Ref	0.80 (0.71,0.89)	0.73 (0.66,0.80)	< 0.001
Model 2	Ref	0.80 (0.71,0.89)	0.73 (0.66,0.81)	< 0.001
Model 3	Ref	0.84 (0.75,0.95)	0.7503 (0.67,0.84)	< 0.001
**Isoflavones**				
No. events	10,870	8,570	9,279	
Mean (SE) (mg/d)	0.00 (0.00)	0.07 (0.00)	5.47 (0.25)	< 0.0001
**OR (95%CI)**				
Model 1	Ref	1.05 (0.94,1.17)	0.69 (0.62,0.76)	< 0.001
Model 2	Ref	1.04 (0.92,1.17)	0.69 (0.62,0.76)	< 0.001
Model 3	Ref	1.09 (0.96,1.23)	0.70 (0.63,0.79)	< 0.001
**Anthocyanidins**				
No. events	9,696	9,371	9,652	
Mean (SE) (mg/d)	0.25 (0.01)	6.51 (0.08)	36.08 (0.94)	< 0.0001
**OR (95%CI)**				
Model 1	Ref	0.81 (0.73,0.90)	0.61 (0.55,0.68)	< 0.001
Model 2	Ref	0.82 (0.74,0.91)	0.62 (0.55,0.70)	< 0.001
Model 3	Ref	0.84 (0.75,0.94)	0.64 (0.57,0.73)	< 0.001

OR, odd ratios; CI, confidence interval. Data are presented as OR and 95% CIs. ORs obtained from average marginal predictions in the logistic analysis model. Model 1 adjusted for age and sex; Model 2 adjusted for age, sex, BMI, smoking status, physical activity, and alcohol consumption; Model 3 adjusted for all covariates in Model 2 plus intakes of energy, protein, carbohydrate, fiber, and total fat. *P*-value based on orthogonal polynomial contrasts of the adjusted prevalence estimates by category of flavonoid (total or subclass) intake.

### Stratified analyses

Results presented that Q3 of total flavonoids intake was significantly related to a lower risk of MetS in participants of age < 20 years (OR = 0.64, 95% CI: 0.50–0.82) compared to Q1, but Q2 of total flavonoids intake was significantly related to a lower risk of MetS in participants of age >60 years (OR = 0.76, 95% CI: 0.58–1.01). Moreover, compared to Q1, Q2, and Q3, total flavonoids intake was significantly related to MetS lower risk in Mexican participants (Q2: OR = 0.78, 95% CI: 0.66–0.92; Q3: OR = 0.79, 95% CI: 0.66–0.95). A negative correlation between total flavonoids intake and MetS prevalence was also found in participants with a BMI of < 25 kg/m^2^. Interaction analysis revealed that different degrees of physical activity had distinct effects on the extent to which total flavonoids consumption affected MetS risk (*P* < 0.0001; Table 3). Compared to Q1, MetS risk was reduced in Q3 of the isoflavones intake (Supplementary material 2), while no significant difference was found in Q2 of the isoflavones intake. Moreover, no interaction existed in the effect of these covariates with isoflavones intake on MetS risk. Additionally, compared to Q1, anthocyanidins and flavanones intake in Q2 and Q3 could significantly lower MetS risk (Supplementary material 3, 4).

**Table 3:** Stratification analysis of the association between total flavonoid intake and MetS.

**Covariates**	**Total population (*n =* 28,719)**				***p* for trend**	***p* for interaction**
**Age**						0.56
≤ 20, *n*	7,804	ref	0.72 (0.57,0.92)	0.64 (0.50,0.82)	< 0.001	
21–39, *n*	6,813	ref	0.94 (0.77,1.13)	0.87 (0.66,1.14)	0.3	
40-59, *n*	7,847	ref	0.96 (0.74,1.24)	0.93 (0.74,1.18)	0.54	
≥60, *n*	6,255	ref	0.76 (0.58,1.01)	0.84 (0.66,1.07)	0.22	
Sex						0.73
Female, *n*	14,690	ref	0.92 (0.80,1.05)	0.99 (0.87,1.13)	0.94	
Male, *n*	14,029	ref	0.86 (0.73,1.00)	0.90 (0.75,1.08)	0.27	
**Ethnicity**						0.19
White, *n*	18,191	ref	0.98 (0.83,1.14)	1.03 (0.88,1.21)	0.63	
Black, n	3,465	ref	0.82 (0.67,1.00)	0.91 (0.73,1.12)	0.37	
Mexican, n	2,970	ref	0.78 (0.66,0.92)	0.79 (0.66,0.95)	0.02	
Other, n	4,093	ref	0.72 (0.56,0.92)	0.77 (0.61,0.96)	0.05	
**BMI (kg/m2)**						0.11
BMI ≥ 25, *n*	12,677	ref	0.98 (0.83,1.15)	0.97 (0.84,1.12)	0.7	
BMI ≤ 25, *n*	16,042	ref	0.74 (0.56,0.97)	0.70 (0.55,0.91)	0.01	
**Smoke**						0.41
No, *n*	24,288	ref	0.90 (0.79,1.03)	0.92 (0.80,1.07)	0.32	
Yes, *n*	4,431	ref	0.90 (0.72,1.12)	1.09 (0.86,1.38)	0.43	
**Alcohol drinking**						
Never, *n*	6,979	ref	0.75 (0.62,0.89)	0.87 (0.69,1.10)	0.3	0.65
Former, *n*	2,257	ref	0.83 (0.63,1.09)	1.00 (0.72,1.39)	0.97	
Mild, *n*	8,587	ref	1.01 (0.76,1.33)	0.94 (0.69,1.26)	0.61	
Moderate, *n*	4,474	ref	0.93 (0.64,1.35)	1.13 (0.83,1.54)	0.35	
Heavy, *n*	6,422	ref	0.92 (0.74,1.15)	0.93 (0.72,1.18)	0.54	
**Physical activity**						
No, *n*	10,399	ref	0.76 (0.65,0.89)	1.16 (1.00,1.35)	0.04	< 0.001
Low, *n*	5,775	ref	0.93 (0.73,1.18)	0.80 (0.62,1.03)	0.07	
Intermediate, *n*	6,304	ref	1.08 (0.86,1.35)	0.82 (0.64,1.05)	0.07	
High, *n*	6,241	ref	0.87 (0.69,1.09)	0.94 (0.71,1.24)	0.73	
**Hypertension**						0.08
No, *n*	20,816	ref	0.79 (0.69,0.91)	0.87 (0.74,1.04)	0.16	
Yes, *n*	7,903	ref	0.99 (0.83,1.19)	0.92 (0.77,1.09)	0.29	
**Diabetic**						0.28
No, *n*	23,972	ref	0.88 (0.75,1.02)	0.92 (0.80,1.06)	0.32	
Yes, *n*	4,747	ref	0.88 (0.71,1.09)	0.86 (0.72,1.04)	0.14	
**CVD**						0.07
No, *n*	26,855	ref	0.91 (0.80,1.03)	0.96 (0.84,1.10)	0.62	
Yes, *n*	1,864	ref	0.71 (0.54,0.94)	0.72 (0.52,0.98)	0.05	
**CKD**						0.002
No, *n*	20,867	ref	0.96 (0.84,1.10)	0.92 (0.80,1.05)	0.21	
Yes, *n*	7,852	ref	0.77 (0.63,0.94)	1.04 (0.84,1.29)	0.6	
**COPD**						0.03
No, *n*	27,645	ref	0.91 (0.80,1.02)	0.94 (0.82,1.07)	0.41	
Yes, *n*	1,074	ref	0.58 (0.38,0.89)	1.07 (0.68,1.69)	0.66	

OR, odd ratios; CI, confidence interval. Data are presented as OR and 95% CIs. ORs were obtained by the logistic analysis with adjusted intakes of energy, protein, carbohydrate, fiber, and total fat. *P*-value based on orthogonal polynomial contrasts of the adjusted prevalence estimates by category of flavonoid (total or subclass) intake. BMI, body mass index; CKD, chronic kidney disease; MET, metabolic equivalent; COPD, chronic obstructive pulmonary disease; CVD, cardiovascular disease; MetS, metabolic syndrome.

### Overall dose–response associations between flavonoids intake and MetS

**Figure 1:**
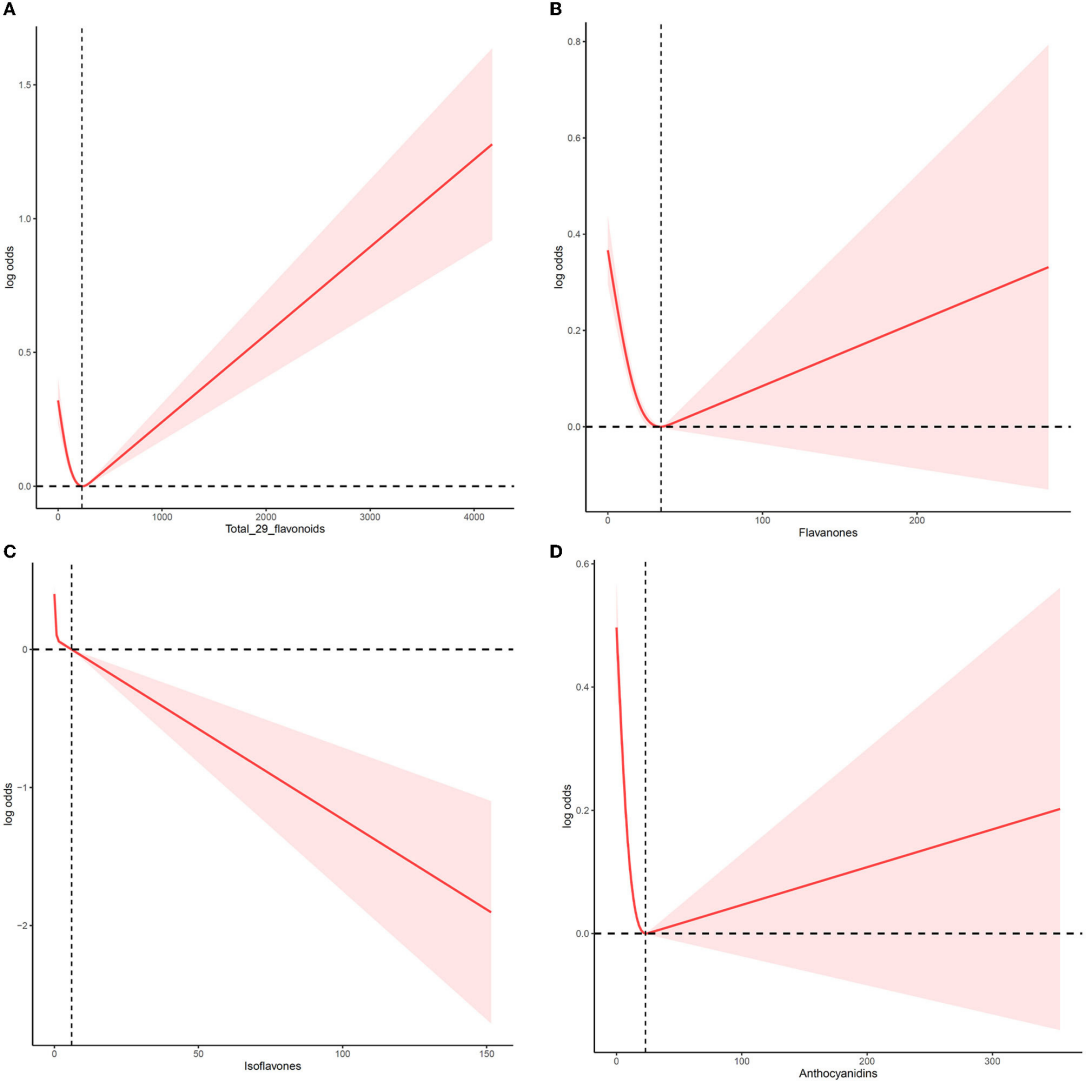
The non-linear trend between the intake of flavonoids and the risk of MetS using a restricted cubic spline. Data are presented as log (odds ratios) (y-axis) and level of flavonoids (mg/d) after adjusted for age, sex, ethnicity, drinking, smoking, physical activity, and other nutrient intake (fiber, fat, protein, and carbohydrate). **(A)** Total flavonoids, **(B)** flavanones, **(C)** isoflavones, and **(D)** anthocyanidins.

We employed an RCS to analyze the dose–response relationship of flavonoid consumption (Figure 1). The median total intake of total flavonoids, flavanones, isoflavones, and anthocyanidins was set as the reference point to illustrate the association between flavonoids intake and MetS reduction. Figure 1A displays a non-linear between flavonoid intake and the risk of MetS after adjusting for age, sex, ethnicity, lifestyle, and other nutrient intakes. MetS prevalence decreased steadily following the increase in total intake of flavonoids until the total flavonoids' intake reached 237.67 mg/day. Then, MetS risk began to increase as the total flavonoids intake evaluate further. Flavanones (Figure 1B) and anthocyanidins (Figure 1D) also displayed V-shaped relationship curves. At 34.37 mg/day (flavanones) and 23.13 mg/day (anthocyanidins) points, MetS risk was the lowest, respectively. In contrast to these flavonoids, when isoflavones intake increased, MetS risk consistently dropped, and the downward trend then slightly increased (Figure 1C). In addition, there was a slight difference between men and women in the dose–response relationship of total flavonoids intake and flavanones intake. As shown in Supplementary Figure 1, the change point of total flavonoids intake was 222 and 237 mg/day for women and men, respectively, and the change point of flavanones intake was 31.09 and 39.09 mg/day for women and men, respectively.

## Discussion

This study first investigated the relationship between total flavonoids and their subclasses intake and the MetS risk in the USA population based on NHANES 2007–2010 and 2017–2018. The results demonstrated that higher total flavonoids intake was significantly related to a lower MetS risk, with 13 and 15% reductions in Q3 and Q2 vs. Q1, respectively. Moreover, this reverse effect of total flavonoids on MetS risk was considerably pronounced in men, younger, Mexican, obese, and CVD individuals. The flavanones, isoflavones, and anthocyanidins intake are also inversely related to MetS risk in any population. Furthermore, the dose–response effect demonstrated a U-shaped curve between the total flavonoids intake (change point at 237.67 mg/day), flavanones (change point at 34.37 mg/day), anthocyanidins (change point at 23.13 mg/day), and MetS risk.

Currently, MetS is an urgent public health problem. Previous epidemiological surveys predicted that the prevalence rate of MetS ranges from 20 to 45%. Additionally, over 85% of adults will be overweight or obese by 2030 (36). Therefore, it is critical to explore effective intervention manner for MetS. Flavonoid-enriched diet and moderate physical activity could be the most effective and less costly manner to improve MetS. A previous study investigated the relationship between flavonoids intake and MetS risk. For instance, higher dietary flavonoid intakes were negatively associated with MetS among Polish individuals (37), Iranian adults (29, 38), and Chinese urban adults (5). In this study, total flavonoids intake, flavanones, isoflavones, and anthocyanidins can also reduce the metabolic syndrome risk, even after adjusting for sex, age, and other factors, which was different from a previous study performed in the Iran population (29). Flavonoids have been associated with other health benefits, including reduced T2D and CVD risk, certain cancers, and neurodegenerative disorders. For example, the Health Professionals Follow-Up Study (1986–2006) reported that higher consumption of anthocyanins and anthocyanin-rich fruit was associated with a lower risk of T2D in the USA population (39). Total flavonoids, anthocyanidins, flavan-3-ols, and flavanones intake were also inversely associated with high CVD and atrial fibrillation (AF) risk (19, 40), whereas intakes of flavones and flavonols were not. Moreover, a high intake of flavones (OR 0.62), flavanones (0.64), and anthocyanins were associated with lower odds of subjective cognitive decline (SCD) (41).

Flavonoids are a large, diverse group of bioactive polyphenolic compounds mainly sourced from fruits, vegetables, cereals, and tea (19, 42). There are six subclasses of flavonoids: anthocyanidins (fruits, particularly berries), flavan-3-ols (tea), flavanones (citrus fruits and juices), flavones (tea, peppers, and celery), flavonols (tea, onions, and potatoes), and isoflavones (soy products) (43–45). An investigation of the main food sources of total polyphenol intake and subclasses between 2008–2009 and 2017–2018 in the Brazilian population showed that coffee was the most significant food source for hydroxycinnamic acids and phenolic acids, contributing with 59.4 and 54.1% to the daily total polyphenols intake (46). Tea, coffee, and fruits are the main sources of flavonoids. Among the population of the USA, flavan-3-ols, primarily derived from tea (94%), comprised 80% of flavonoids intake (47). It has been found that consumption of certain foods, such as blueberries, strawberries, apples, orange juice, grapefruit juice, bananas, onions, tea, and peaches, could independently predict the development of SCD in the future (41). However, future multiple-center longitudinal studies must confirm the causal relationship between each flavonoid intake and metabolic syndrome and other diseases.

Indeed, earlier research specified that flavonoids improved metabolic syndrome through their antioxidant and anti-inflammatory properties to repair endothelial function and enhance nitric oxide (NO) bioavailability (48, 49). For example, naringenin, one flavanone compound, can downregulate the levels of triglyceride (TG) and phospholipid and increase the gene expression of PPAR-α, CPT-1, and uncoupling protein (UCP)-2 to reduce blood lipid (50, 51). It could also activate the peroxisome proliferator-activated receptor (PPAR) and adiponectin expression and decrease the liver X receptors (LXR)-α level (52, 53) to treat adiposity and atherosclerosis. Moreover, naringenin showed strong anti-inflammation activity by inhibiting NF-κB activation, the levels of myeloperoxidase (MPO), N-acetyl-β-D-glucosaminidase (NAG), TNF-α, interleukin (IL)-1β, IL-6, and IL-12 (54), and involved into the NO-cGMP-PKG-KATP signaling pathway (36). Therefore, naringenin can potentially improve MetS, which aligns with our findings that flavanones may lower the risk of MetS. Isoflavones, like genistein and puerarin, may influence insulin release and lipids metabolism by blocking adipocyte-specific proteins and controlling PPAR-γ levels (55–57). Additionally, consuming anthocyanins, such as pelargonidin, cyanidin, delphinidin, peonidin, and malvidin, can help treat the pathology of MetS and disorders linked to MetS by reducing body weight, insulin resistance, inflammation, and oxidative stress injury (36). Recent studies have found that dietary lifestyle can affect the structure of the gut microbiome and its metabolites, thereby influencing the development of MetS. By altering the host gut flora, resveratrol, for instance, could decrease body weight and body fat to improve glucose homeostasis and obesity (58). Future randomized clinical trials should be designed to confirm these potential mechanisms in multiple districts.

Although flavonoid intake effectively attenuated MetS, RCS curves showed complex non-linear relationships between flavonoids intake and MetS risk rather than monotonic increasing or decreasing relationships. Additionally, the RCS curves of flavonoids consumption showed slight differences for men and women, indicating that the prevalence of MetS and the amount of flavonoids intake varied by gender. Indeed, men had a significantly higher intake of flavanones (citrus) and flavonols (mixed dishes and beer) than women. Women had a significantly higher intake of anthocyanidins (berries) compared with men (47). Moreover, our previous study found that a diet of 7,8-dihydroxyflavone (7,8-DHF) could protect the function of the female hypothalamic–pituitary–ovarian (HPO) axis and activate tissue-specific ERα to maintain body metabolic homeostasis (59). Additionally, intake rates of flavonoids varied by geographic region, dietary preferences, sociodemographic characteristics, and lifestyle choices. Previous studies reported that the mean flavonoids intake was 34.68 mg/day in Chinese (5), whereas the mean flavonoids intake was 189.7 mg/day in 1999–2002 (60) in the U.S. population. Future large-scale and multi-center clinical trials should be conducted to establish safe doses and create an individual's healthcare program for the potential health implications of attuning the risk of MetS.

The strength of this study was that it was the first large sample, population-based, cross-sectional study that reported the effect of flavonoid intake and its subclasses on MetS based on NHANES from 2007–2010 to 2017–2018. The effects of total flavonoids, flavanones, isoflavones, and anthocyanidins consumption on MetS were evaluated, providing diet recommendations for people in the USA. However, this study has several limitations. First, this study is a cross-sectional investigation that could only present relationships between flavonoids and MetS. Second, flavonoid data was obtained by the 24-h recall, whereas MetS might have already occurred before the interview. Therefore, a bias in the effects of flavonoids intake on MetS is unavoidable. Third, the participants included in this study were all Americans. Consequently, the effects of flavonoids intake on MetS observed in this study may be unsuitable for Asians or other populations.

## Conclusion

Higher flavonoid intakes could reduce MetS risk in the USA population. In addition, flavanones, isoflavones, and anthocyanidins are the most effective flavonoid subclasses in attenuating MetS, while other flavonoid subclasses showed a relatively small effect. Total flavonoids, flavanones, isoflavones, and anthocyanidins demonstrated non-linear relationships with MetS risk. The most effective doses of total flavonoids, flavanones, and anthocyanidins were 237.67, 34.37, and 23.13 mg/day, respectively. Further large-scale randomized controlled trials should be performed to establish causality between flavonoids intake and MetS risk and the safe doses of flavonoids in different populations. Regarding the perspective of public health, our findings may provide fresh insight into MetS risk based on flavonoids intake and build future tailored dietary recommendations as a preventative tool against metabolic syndrome based on the most effectively calculated amounts.

## Data availability statement

The raw data supporting the conclusions of this article will be made available by the authors, without undue reservation.

## Author contributions

ZZ and WX: conceptualization and writing—review and editing. WG and XD: methodology. WG and CX: software. GM and CX: validation. ZZ: formal analysis. XX: data curation. GM and WX: writing—original draft preparation. All authors have read and agreed to the published version of the manuscript.
