# Supplementary material for: The association between dietary intake of flavonoids and its subclasses and the risk of metabolic syndrome
Source: Front Nutr. 2023 Jul 5;10:1195107. doi: 10.3389/fnut.2023.1195107 (PMC10354435; doi:10.3389/fnut.2023.1195107)
Supplement: Supplementary file 1 [file Table_1.docx]

**Supplemental Table 1. Characteristics of NHS Participants by Quintiles of Total Flavonoid Intake**

|  | **Total population** | **Total flavonoid intake quintiles** | | |  |
| --- | --- | --- | --- | --- | --- |
|  |  | **Q1** | **Q2** | **Q3** | **P value** |
| **Total_29_flavonoids** | **186.08(4.77)** | **18.63(0.25)** | **92.68(0.84)** | **410.71(9.99)** | **< 0.0001** |
| **Isoflavones** | **1.79(0.09)** | **0.00(0.00)** | **0.07(0.00)** | **5.47(0.25)** | **< 0.0001** |
| Daidzein | 0.69(0.03) | 0.00(0.00) | 0.04(0.00) | 2.12(0.10) | < 0.0001 |
| Genistein | 0.95(0.05) | 0.00(0.00) | 0.03(0.00) | 2.92(0.14) | < 0.0001 |
| Glycitein | 0.14(0.01) | 0.00(0.00) | 0.01(0.00) | 0.42(0.02) | < 0.0001 |
| **Anthocyanidins** | **14.34(0.44)** | **0.25(0.01)** | **6.51(0.08)** | **36.08(0.94)** | **< 0.0001** |
| Cyanidin | 2.48(0.11) | 0.07(0.00) | 1.19(0.01) | 6.29(0.32) | < 0.0001 |
| Delphinidin | 1.57(0.08) | 0.00(0.00) | 0.37(0.01) | 4.38(0.22) | < 0.0001 |
| Malvidin | 5.47(0.17) | 0.00(0.00) | 1.46(0.02) | 15.67(0.39) | < 0.0001 |
| Pelargonidin | 1.63(0.09) | 0.00(0.00) | 0.23(0.00) | 4.85(0.23) | < 0.0001 |
| Peonidin | 1.79(0.11) | 0.00(0.00) | 0.37(0.00) | 5.05(0.29) | < 0.0001 |
| Petunidin | 1.12(0.06) | 0.00(0.00) | 0.28(0.01) | 3.18(0.17) | < 0.0001 |
| **Flavan_3_ols** | **140.43(4.45)** | **4.12(0.05)** | **39.23(0.88)** | **351.61(9.93)** | **< 0.0001** |
| Catechin | 7.30(0.12) | 1.42(0.02) | 6.08(0.03) | 13.50(0.22) | < 0.0001 |
| Catechins | 62.88(1.97) | 4.03(0.05) | 26.00(0.38) | 149.97(4.87) | < 0.0001 |
| Epicatechin | 9.79(0.16) | 1.47(0.02) | 7.94(0.04) | 18.97(0.34) | < 0.0001 |
| Epicatechin_3_gallate | 8.50(0.31) | 0.00(0.00) | 1.42(0.05) | 22.57(0.75) | < 0.0001 |
| Epigallocatechin | 13.28(0.48) | 0.06(0.00) | 2.35(0.09) | 34.92(1.22) | < 0.0001 |
| Epigallocatechin_3_gallate | 22.48(0.92) | 0.00(0.00) | 3.25(0.18) | 60.69(2.38) | < 0.0001 |
| Gallocatechin | 1.35(0.05) | 0.00(0.00) | 0.30(0.01) | 3.53(0.10) | < 0.0001 |
| Theaflavin | 1.27(0.05) | 0.00(0.00) | 0.44(0.02) | 3.51(0.11) | < 0.0001 |
| Theaflavin_3_3_digallate | 1.40(0.05) | 0.00(0.00) | 0.54(0.02) | 3.94(0.12) | < 0.0001 |
| Theaflavin_3_gallate | 1.01(0.04) | 0.00(0.00) | 0.54(0.01) | 2.87(0.09) | < 0.0001 |
| Theaflavin_3q_gallate | 1.19(0.05) | 0.00(0.00) | 0.47(0.01) | 3.45(0.11) | < 0.0001 |
| Thearubigins | 72.79(2.63) | 0.00(0.00) | 40.69(0.67) | 195.97(5.73) | < 0.0001 |
| **Flavanones** | **13.83(0.29)** | **0.06(0.00)** | **7.34(0.15)** | **36.55(0.50)** | **< 0.0001** |
| Eriodictyol | 0.26(0.01) | 0.00(0.00) | 0.08(0.00) | 0.79(0.02) | < 0.0001 |
| Hesperetin | 9.91(0.19) | 0.00(0.00) | 5.39(0.11) | 27.17(0.39) | < 0.0001 |
| Naringenin | 3.25(0.11) | 0.04(0.00) | 1.42(0.03) | 8.72(0.23) | < 0.0001 |
| **Flavones** | **0.78(0.02)** | **0.10(0.00)** | **0.51(0.00)** | **1.54(0.04)** | **< 0.0001** |
| Apigenin | 0.18(0.01) | 0.01(0.00) | 0.08(0.00) | 0.42(0.03) | < 0.0001 |
| Luteolin | 0.61(0.01) | 0.07(0.00) | 0.39(0.00) | 1.23(0.03) | < 0.0001 |
| **Flavonols** | **15.68(0.23)** | **4.29(0.03)** | **11.65(0.04)** | **26.85(0.33)** | **< 0.0001** |
| Isorhamnetin | 0.70(0.01) | 0.05(0.00) | 0.41(0.00) | 1.44(0.02) | < 0.0001 |
| Kaempferol | 3.74(0.06) | 0.48(0.00) | 2.20(0.01) | 7.26(0.11) | < 0.0001 |
| Myricetin | 1.27(0.03) | 0.12(0.00) | 0.68(0.00) | 2.63(0.05) | < 0.0001 |
| Quercetin | 9.95(0.14) | 2.91(0.03) | 7.86(0.02) | 16.90(0.21) | < 0.0001 |

Note: the values of flaviods intake were expressed as mean and SE. Bold font represents six subclasses of total flavonoid compounds, and the non-bold font beneath each bold font indicates its compounds.
