# Supplementary material for: The association between dietary intake of flavonoids and its subclasses and the risk of metabolic syndrome
Source: Front Nutr. 2023 Jul 5;10:1195107. doi: 10.3389/fnut.2023.1195107 (PMC10354435; doi:10.3389/fnut.2023.1195107)
Supplement: Supplementary file 5 [file Data_Sheet_1.docx]

**Supplementation Figure 1**


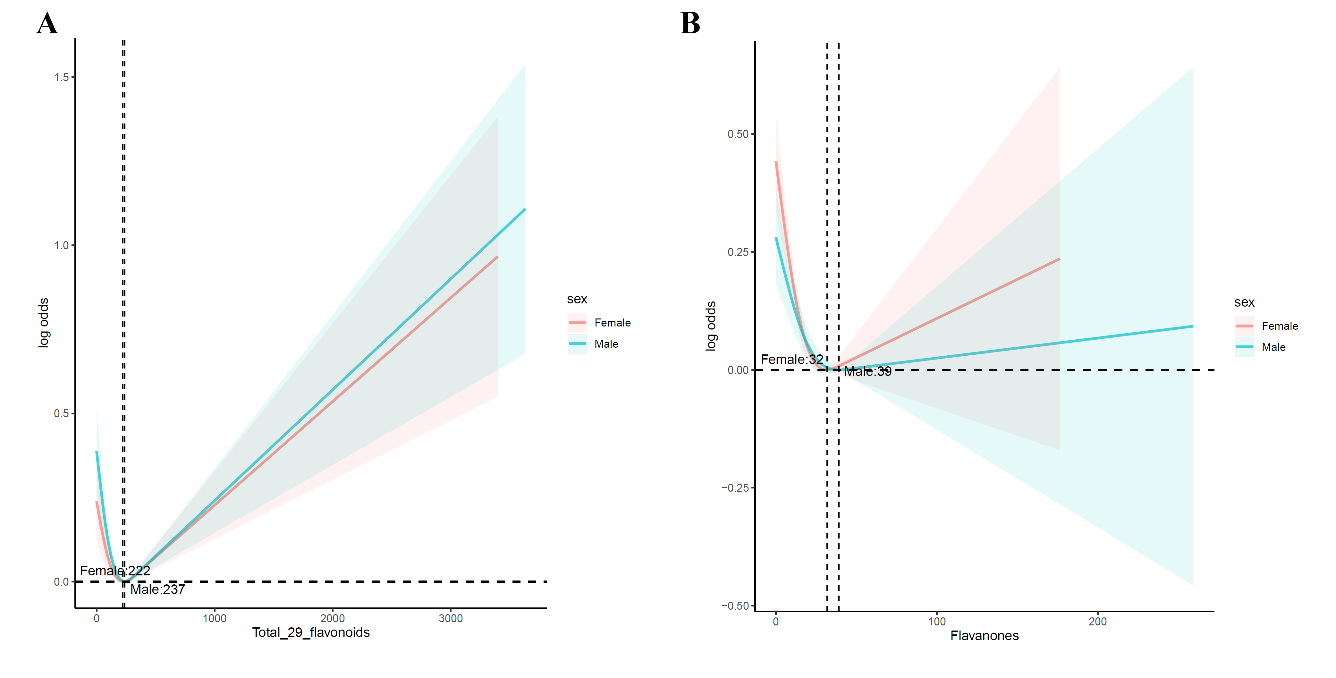


**Supplementation Figure 1. The non-linear trend between the intake of total flavonoids and Isoflavones and the risk of MetS in male and female population.** Data are presented as log (odds ratios) (y axis) and level of flavonoids (mg/d). (A) Total flavaoniods, (B) Isoflavones.
